# Components and Outcomes of Internet-Based Interventions for Caregivers of Older Adults: Systematic Review

**DOI:** 10.2196/jmir.7896

**Published:** 2017-09-19

**Authors:** Cassioppée Guay, Claudine Auger, Louise Demers, W Ben Mortenson, William C Miller, Dominique Gélinas-Bronsard, Sara Ahmed

**Affiliations:** ^1^ School of Rehabilitation Faculty of Medicine Université de Montréal Montreal, QC Canada; ^2^ Centre for Interdisciplinary Research in Rehabilitation of Greater Montreal Montreal, QC Canada; ^3^ Centre de recherche de l'Institut universitaire de gériatrie de Montréal Montreal, QC Canada; ^4^ Department of Occupational Science and Occupational Therapy Faculty of Medicine University of British Columbia Vancouver, BC Canada; ^5^ GF Strong Rehabilitation Center Vancouver, BC Canada; ^6^ International Collaboration on Repair Discoveries Vancouver, BC Canada; ^7^ School of Physical and Occupational Therapy Faculty of Medicine McGill University Health Centre, Clinical Epidemiology, McGill University Montreal, QC Canada

**Keywords:** systematic review, caregivers, aged, Internet-based interventions, Internet, behavior change

## Abstract

**Background:**

When trying to access interventions to improve their well-being and quality of life, family caregivers face many challenges. Internet-based interventions provide new and accessible opportunities to remotely support them and can contribute to reducing their burden. However, little is known about the link existing between the components, the use of behavior change techniques, and the outcomes of these Internet-based interventions.

**Objective:**

This study aimed to provide an update on the best available evidence about the efficacy of Internet-based interventions for caregivers of older adults. Specifically, the components and the use of behavior change techniques and how they impact on the efficacy of the intervention were sought.

**Methods:**

A systematic review searched primary source studies published between 2000 and 2015. Included studies were scored with a high level of evidence by independent raters using the GRADE criteria and reported caregiver-specific outcomes about interventions delivered through the Internet for caregivers of people aged 50 years and older. A narrative synthesis identified intervention components (eg, content, multimedia use, interactive online activities, and provision of support), behavior change techniques, and caregiver outcomes (eg, effects on stressors, mediators, and psychological health). The risk of bias within the included studies was assessed.

**Results:**

A total of 2338 articles were screened and 12 studies describing 10 Internet-based interventions were identified. Seven of these interventions led to statistically significant improvements in caregiver outcomes (eg, reducing depression or anxiety, n=4). These efficacious interventions used interactive components, such as online exercises and homework (n=4) or questionnaires on health status (n=2) and five of them incorporated remote human support, either by professionals or peers. The most frequently used behavior change techniques included in efficacious interventions were provision of social support (n=6) and combinations of instructions to guide behavior change and barrier identification (n=5). The design and aim of the included studies did not permit determining exactly which component and/or behavior change technique was more efficacious in producing positive outcomes in caregivers. The risk for selection bias was low for all the studies, and low to high for performance, detection, and attrition biases.

**Conclusions:**

In sum, Internet-based interventions that incorporate professional and social support, and provide instructions to change behavior and problem solve in an interactive manner appear to lead to positive outcomes in caregivers. Studies isolating the specific effect of components are needed to improve our understanding of the underlying mechanism of action.

## Introduction

A family caregiver (henceforth described as caregivers) is a person who provides care without any financial compensation for a family member, a friend, or a loved one with long-term health problems or disabilities [1]. Approximately 5.4 million Canadians [2] and 34.2 million Americans [3] are caregivers of older adults. Aging and associated frailty are the main causes of caregiving needs [2,3], which are expected to increase because of population aging in Western countries. Moreover, older adults are more prone to experience disability related to long-term physical conditions and memory problems [3,4], which altogether increase the caregiver burden. The term “burden” is used to describe the harmful physical, emotional, and social effects caregivers may experience [5]. Role overload and burden put caregivers at higher risk for depression, anxiety, and negative levels of stress [6,7]. In 2012 in Canada, one in two caregivers of an older adult felt anxious about their responsibilities and one in six experienced depressive symptoms [8]. In the United States, 17% of the caregivers of older adults report poor or fair health and one in five report a decrease in their health due to caregiving [3].

### Stress Process Model

The Stress Process Model [9] is a conceptual framework that is used to explicate the stress and burden experienced by caregivers. Caregiving may produce primary stressors, which are situations that are perceived as problematic or harmful by the caregiver. These stressors can be described objectively (eg, low level of independence of the care recipient, observable behaviors) and subjectively (eg, feelings of overload and relational deprivation). If stressors persist, they may lead to secondary role strains (eg, job-caregiving conflicts, constriction of social life) and intrapsychic strains (eg, decreased self-esteem and sense of mastery, loss of self). Primary stressors and secondary strains contribute to overall caregiver burden, which can result in adverse outcomes on psychological and physical health as well as on social participation. According to the Stress Process Model, personal resources, such as coping strategies and access to social support, are mediators of the stress process that can help mitigate adverse outcomes. The model also acknowledges that caregiving can lead to positive effects, such as a sense of inner growth while facing challenges.

Considering the stress and burden they experience, caregivers of older adults require health care, psychosocial, community, and respite services to prevent negative outcomes related to their caregiving role. However, caregivers experience many barriers when trying to obtain those services, such as lack of transportation to access the intervention, unavailability of a secondary caregiver to take over in their absence, and lack of flexibility to participate in a highly demanding intervention [10]. Consequently, programs that are delivered outside of the home setting have been shown to be less attended to by caregivers than home-based programs, such as telephone counseling, home visits, and technology-based interventions [10]. Internet-based interventions can thus offer an easily accessible alternative [11-17] and can be more cost-effective than traditional face-to-face interventions [18].

### Internet-Based Interventions for Caregivers

Internet-based interventions, also referred to as eHealth interventions or information and communication technology-based interventions, are defined as therapeutic programs with specific health objectives delivered mainly using the Internet [19]. They have been classified by Barack and Klein [19] into six categories: Web-based education interventions, self-help Web-based therapeutic interventions, human-supported Web-based therapeutic interventions, online counseling, Internet-operated therapeutic software, and other online activities. Each category of Internet-based intervention is described with respect to four major types of components: (1) content (eg, educational or aimed for behavior change), (2) multimedia (eg, text, images, videos), (3) interactive online activities (eg, online quizzes, homework), and (4) guidance and supportive feedback (eg, automatic reminders, professional feedback).

To our knowledge, seven reviews [11-17] have described the efficacy of Internet-based interventions for caregivers of older adults, but the portrait they provide is incomplete regarding the quality of the evidence and the components driving the success of the interventions. The reviews included Internet-based interventions for caregivers of all ages [12,13] or caring for people with cancer [14], dementia [11,17], for community-dwelling older adults [15], or for adults and older adults with intellectual disabilities [16]. The reviews report that Internet-based interventions can effectively reduce depression and caregiver burden [11-14,16,17], as well as having positive effects on caregivers’ sense of competence/self-efficacy [11,17], coping strategies [14], knowledge about the care recipient’s condition [17], and quality of life [14]. Lastly, the review by Magnusson et al [15] gave interesting insights on success factors and feasibility of Internet-based interventions, universal design principles, and older adults’ thoughts and attitudes toward technology, but not on the efficacy of included interventions. Concerning methodological aspects, only two reviews produced a complete analysis of the quality of evidence, which was deemed poor [11] or acceptable [13]. Other reviews either described the quality of the evidence using scales [12,14], but did not use this assessment to critically appraise the strength of reported results or did not report anything on the study designs or risk of bias [15-17]. None of the seven reviews used a framework to classify the components, thus making it difficult to compare components of efficacious Internet-based interventions and to identify hypothetical causes of efficacy. In sum, the currently available reviews indicate that Internet-based interventions for caregivers of older adults facing different health conditions have the potential to generate positive outcomes on psychological health. The strength of evidence of these results, however, is uncertain and reasons of observed improvements in caregivers’ outcomes were poorly documented and not reported uniformly.

### Behavior Change Techniques in Internet-Based Interventions

One factor that might explain the efficacy of Internet-based interventions, aside from the components of the intervention itself, is the incorporation of behavior change techniques (BCTs). BCTs are strategies that promote behavior change by, for example, providing information on consequences of behavior on health, prompting users to identify barriers to behavior change or offering social support. A review by Webb et al [20] found that Internet-based interventions promoting healthy living habits that are theoretically grounded and using BCTs are associated with larger effect sizes when compared with another intervention or no intervention at all. Similarly, a rapid review on the potential of Internet-based interventions for self-management argues that the incorporation of cognitive-behavioral therapy as well as BCTs is required to attain certain behavioral outcomes (eg, healthier living habits, safer sex) [21]. Given the encouraging results of integrating BCTs in the fields of health promotion and prevention for care recipients, the investigation whether Internet-based interventions for caregivers should enhance self-management by encouraging the development of new behaviors with the use of BCTs is relevant. For this, the taxonomy by Abraham and Michie [22], which proposed a series of BCTs to incorporate into interventions aiming to support behavior change, can be used to identify and classify BCTs as a way of pinpointing underlying mechanisms of effects. This has not been done in any previous reviews for caregivers of older adults to our knowledge.

### Aim and Specific Objectives

This study aimed to provide an update on the best available evidence about the efficacy of Internet-based interventions for caregivers of older adults. Specific objectives were to (1) classify the components that are found in Internet-based interventions for caregivers of older adults, (2) describe the BCTs used in these Internet-based interventions, and (3) explore which intervention components and BCTs of Internet-based interventions are associated with efficacious outcomes in caregivers.

## Methods

### Information Sources and Search Method

Following the Preferred Reporting Items for Systematic Reviews and Meta-Analyses (PRISMA) statement [23], a systematic literature search was conducted in MEDLINE, EMBASE, and CINHAL, covering studies published from January 2000 to July 2015. With the help of a biomedical research librarian, a list of more than 50 medical subject headings, descriptors, and keywords, matched to the specific thesaurus of every database consulted, was used to identify the population (caregivers) and the interventions (Internet-based) of interest (see Multimedia Appendix 1). In addition, we used keywords related to the concepts of self-management and behavior change to target interventions with BCTs for achieving outcomes on the health and well-being of caregivers.

### Eligibility Criteria

Studies were included if they (1) were original papers published in peer-reviewed journals, (2) reported on an intervention of which the principal mode of delivery was the Internet, (3) reported caregiver-specific outcomes, (4) targeted caregivers of older adults and thus had a sample including at least one caregiver of a person aged 50 years or older, and (5) were of a high level of evidence according to the Grading of Recommendations Assessment, Development and Evaluation (GRADE) criteria [24]. As per these criteria, the initial level of evidence was determined based on study designs (eg, high level for randomized controlled trials, moderate level for quasi-randomized controlled trials, and low levels for observational studies). Studies were scored lower if a combination of serious limitations were present (eg, lack of allocation concealment, lack of blinding, incomplete accounting of patients and outcomes events, selective reporting, and other limitations). Studies were scored higher than their original level if they displayed strong methodological qualities (eg, no plausible cofounders, no major treats to the validity of the results), if a dose-response gradient was found, or if the effect would have been reduced by cofounders. Studies were excluded if they were not written in either French or English. Descriptive studies analyzing data consisting of Internet transcripts (eg, messages on a forum, blogs, posting) were also excluded.

### Study Selection

**Figure 1:**
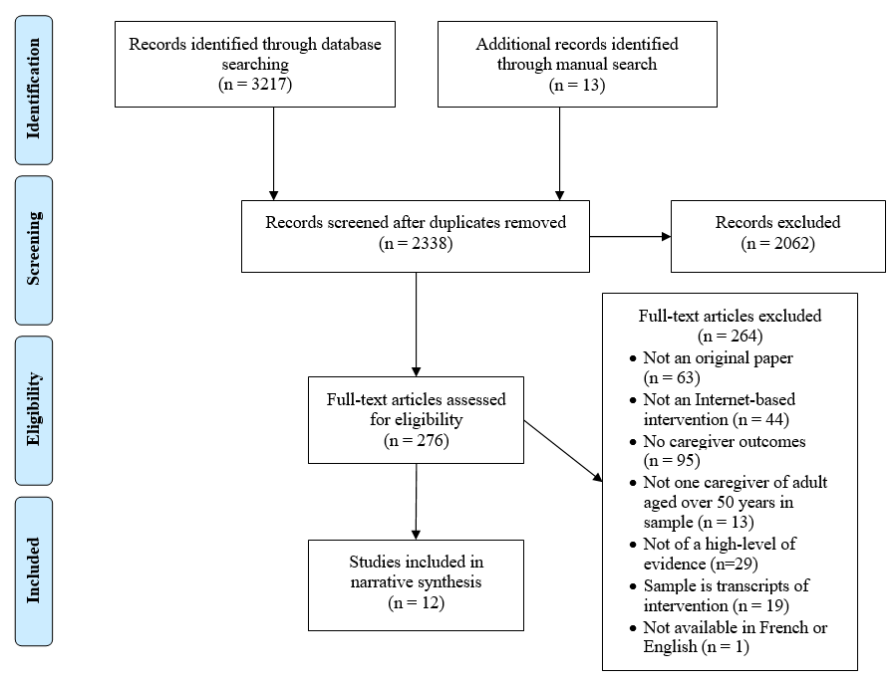
PRISMA flowchart of the search strategy and results.

The complete selection process is detailed in Figure 1. Titles and abstracts were reviewed by the first author. Full-text articles were obtained for relevant studies and the preceding criteria were applied. When the first author was uncertain about the inclusion of studies, they were reviewed independently by a second author (DGB). If consensus could not be reached, discrepancies were resolved with the help of a third author (CA). A manual search was conducted in the reference lists of reviews found during this search.

### Data Collection and Coding

The first author abstracted data from included studies using Excel forms to record the study characteristics, components of the Internet-based interventions, and the use of BCTs. Caregiver population, experimental and control conditions, data collection for reported outcomes, analyses performed, and additional characteristics specific to Internet-based trials as outlined in the CONSORT-EHEALTH (Consolidated Standards of Reporting Trials) guidelines (eg, computer literacy, intended dosage, usage outcomes) [25] were documented.

Caregiver outcomes were classified with the Stress Process Model [9] to outline which factors of the stress process were measured: primary stressors, secondary role strains, secondary intrapsychic strain, or outcomes.

All Internet-based interventions and their components were coded using the Barack and Klein categorization [19]. Following this classification, interventions were classified in one of six possible categories. First were Web-based education interventions, which are online programs or websites providing general standardized content to improve knowledge, awareness, and understanding of users, but not their behavior. Second and third were self-help Web-based therapeutic interventions and human-supported Web-based therapeutic interventions, which are both multicomponent online interventions with content tailored to support behavior change. Those two distinguish themselves by the amount of guidance and feedback they provide: self-help interventions are designed to be self-guided by the user and so do not require interactions with humans, whereas human-supported interventions provide a variety of support means from professionals or peers. Online counseling is the fourth category and refers to programs or technologies that primarily enable professional-user communication for remote counseling, usually on subjects such as mental health or psychological follow-ups. Within the fifth category were Internet-operated therapeutic software, which are programs or devices that use advanced computer capabilities (eg, artificial intelligence principles, augmented realities, or algorithms) to produce a robotic therapist simulation providing dialog-based therapy with patients, rule-based systems/games (eg, WII console), or three-dimensional environments (eg, Second Life). Finally, the category of other online activities consisted of any other websites, blogs, informal support groups, wikis, podcasts, and self-assessments available on the Web that did not have a specified therapeutic goal. Components found from the description of interventions provided by the authors in the article were classified within the four major component categories, as described in Textbox 1.

**Components of Internet-based interventions (adapted from Barack and Klein [19]).**

Content: nature of the information disseminated through the program. Can be generic and educative or designed to create a therapeutic change.

Multimedia: means used to disseminate the content (eg, text, graphics, video).

Interactive online activities: opportunities given to participate actively within the program (eg, quizzes, exercises, questionnaires).

Guidance and supportive feedback: tools by which users access external information about their performance and progress. Can be offered automatically with integrated algorithms (eg, reminders) or by professionals and/or peers through asynchronous (eg, email, forums, bulletin boards) or synchronous (eg, videoconference) components.

Behavior change techniques were also extracted from the description of interventions found in the report using the taxonomy of Abraham and Michie [22]. This taxonomy details 26 BCTs that can be incorporated in interventions, ranging from the simple provision of information on consequences of one’s behavior to more complex techniques such as providing complete goal setting, modeling appropriate behavior, or providing detailed feedback on behavior performance.

Finally, although a high level of evidence per the GRADE criteria was a condition for inclusion, risk of bias was still assessed as “high,” “low,” or “unclear” for the random sequence generation and concealment of allocation (selection bias), for blinding of outcome assessors and the use of valid measures (detection bias), for the blinding of the participants (performance bias), and for how withdrawals were statistically accounted for (attrition bias). This was done as recommended by the Cochrane Handbook for Systematic Reviews of Interventions [26]. Given the difficult nature of double blinding in psychosocial and self-management intervention trials, which comprise the majority of Internet-based interventions for caregivers [12], the double-blind criterion was not assessed.

## Results

After applying the search strategy detailed in Figure 1, a total of 12 studies were retained for a narrative synthesis [27-38]. Records were excluded mainly because they were not a primary source study, they were not delivered through the Internet, or they did not report caregiver-specific outcomes. Twenty-nine studies were excluded because of the quality of their study design; per the GRADE criteria, 8 were of a moderate level of evidence [39-46], 18 of a low level [47-64], and 3 were of a very low level [65-67]. Three reports were downgraded from a high level of evidence to a moderate level because of high differential rates of dropouts between control and experimental conditions that were not accounted for with proper intention-to-treat analysis [40,42,43]. None of the studies were upgraded.

### Characteristics of the Included Studies

The main characteristics of the included studies are presented in Tables 1 and 2. Regarding the studied population, caregivers were mainly female adults or older adults and generally were either a spouse or a child of the care recipient. They cared for people with dementia [27,28,30,38], stroke [32,33,36,37], cancer [29,31,35], or traumatic brain injury [34].

**Table 1:** Characteristics of the study population of included studies (N=12).

Author	Country	N	Mean age (SD)^b^	Female^b^	Relationship with care recipient^b^	Diagnosis of care recipient^b^
Beauchamp et al [27]	USA	299	46.9 (12.2)	73%	Child (67%)	Dementia
Blom et al [28]	Netherlands	245	61.2 (12.37)	69.4%	Spouse (58.4%)	Dementia
Chih et al [29]	USA	235	56	64.2%	Spouse/partner (69.3%)	Cancer
Cristancho-Lacroix et al [30]	France	49	64.2 (10.3)^a^	16 (64%)^a^	Child (64%)^a^	Dementia
DuBenske et al [31]	USA	246	55.56	68.3%	Spouse/partner (72%)	Cancer
Eames et al [32]	Australia	61	55.5	64%	Spouse/partner (67%)	Stroke
Kim et al [33]	South Korea	36	53 (13.7)	NR	Spouse (66.7%)^a^	Stroke
McLaughlin et al [34]	USA	201	NR, 34.6% aged 51-60^a^	86.4%^a^	NR	TBI
Namkoong et al [35]	USA	285	55.56	68.3%	NR	Cancer
Pierce et al [36]	USA	103	54 (12.2)^a^	69.4%^a^	Wife (41.7%)^a^	Stroke
Smith et al [37]	USA	32	55.3 (6.9)^a^	100%	Wife (100%)	Stroke
Torkamani et al [38]	UK, Spain, and Greece	60	60.69 (13.90)	45%	NR	Dementia

^a^For intervention group only.

^b^NR: none reported; SD: standard deviation; TBI: traumatic brain injury.

**Table 2:** Description of the intervention and control groups of included studies (N=12).

Author	Intervention				Control group	
	n^a^	Duration^b^	Description	n^a^		Description
Beauchamp et al [27]	150	30 days	Caregiver’s Friend: Dealing with Dementia—an ongoing worksite Web-based support program providing materials tailored to the needs of caregivers in 3 distinct modules (being a caregiver, coping with emotions, and common difficulties)	149		Usual care wait list
Blom et al [28]	149	5-6 months	Mastery Over Dementia: a 9-lesson online program; the first 8 lessons followed the same sequence: provision of information, exercises, homework, and feedback; lessons were about coping with behavioral problems, relaxation, arranging help from others, changing nonhelping thoughts, and communication; final lesson was a recap and booster session	96		E-bulletin sent by email every 3 weeks for 6 months; content did not overlap with intervention
Chih et al [29]	118	12-24 months	Comprehensive Health Enhancement Support System (CHESS): a password-protected website in which users self-directed to a variety of services (information, communication, and coaching); content covered cancer, caregiving and palliative care, emotional distress, use of coping techniques, and communication techniques	117		Access to the same intervention, without one component (clinical report) for 12-24 months
Cristancho-Lacroix et al [30]	25	12 weeks^c^	Diapason: a password-protected website offering information, skills training and a forum for caregivers; content was divided in 12 thematic sessions with videos covering caregiver stress, understanding the disease, maintaining the loved ones’ autonomy, understanding their reactions, coping with behavioral and emotional troubles, communicating, improving their daily lives, avoiding falls, pharmacological and nonpharmacological interventions for caregivers, social and financial support, and about the future	24		Usual care
DuBenske et al [31]	124	2 years or up to 13 months after the death of the care recipient^c^	Comprehensive Health Enhancement Support System (CHESS): a password-protected website in which users self-directed to a variety of services (information, communication, and coaching); content covered cancer, caregiving and palliative care, emotional distress, use of coping techniques, and communication techniques	122		Access to a list of cancer and palliative care websites constructed from the opinions of clinicians in addition to usual care
Eames et al [32]	31	3 months^c^	What You Need to Know About Stroke: an educational package online containing a list of 34 topics regarding stroke; the Web-based intervention was reinforced with 3 face-to-face and 3 telephone meetings with participants	30		Usual care for the care recipient
Kim et al [33]	18	9 weeks	A Web-based program incorporating education and resources to support self-efficacy in the home setting. Content was divided in nine video sessions covering three themes: understanding stroke, recurrence prevention, and family life	18		Access to an e-bulletin over the course of 6 months
McLaughlin et al [34]	104	3 months	Brain Injury Partner: a Web-based program designed to improve family advocacy skills with content covering advocacy skills, strategies for reducing stress, and to determine necessary professional support needs	97		Access to the Brain Injury Association of America (BIAUSA)
Namkoong et al [35]	141	2 years	Comprehensive Health Enhancement Support System (CHESS): a password-protected website in which users self-directed to a variety of services (information, communication, and coaching); content covered cancer, caregiving and palliative care, emotional distress, use of coping techniques, and communication techniques	144		Access to a list of high-quality patient-directed cancer and palliative care websites in addition to usual care
Pierce et al [36]	51	1 year	Caring-Web: an educational and support intervention that answered questions, discussed options, and gave up-to-date information covering frequently requested topics like stroke disease process, safe transfer techniques, and emotional changes	52		Specific instructions to not buy or use Internet during the study in addition to usual care
Smith et al [37]	15	11 weeks	A Web-based conferencing and video education intervention designed to provide the caregiver with knowledge, resources, and skills; content was divided in 9 weekly video topics covering how to get in touch with your feelings as a caregiver, understanding what it’s like to be a care recipient, being a good listener, nonverbal behavior, choice/control/predictability, relaxation and positive imagery to control stress, and the role of pleasant activities	17		Access only to one component of the intervention that presents links to resources
Torkamani et al [38]	30	6 months	A technology pLatform for the Assisted living of Dementia elDerly Individuals and their carers (ALADDIN): a Web-based program designed to provide support and information with content covering dementia and relaxation/exercises techniques	30		No attention or intervention given

^a^Before attrition.

^b^Length of access to intervention.

^c^In addition to usual care.

One Internet-based intervention was designed specifically to answer the needs of caregivers who were also workers [27]. None of the other studies mentioned the working status of caregivers. Three studies [29,31,35] assessed caregiver comfort levels with using the Internet on a five-point scale ranging from not comfortable to extremely comfortable. On the whole, caregivers rated themselves as being somewhat comfortable with using the Internet (mean scores ranging from 2.36 to 2.54). One study enrolled only novice users of the Internet [36].

The setting of the interventions varied across the included studies in terms of dosage, comparison conditions, and reported adherence. Half of the Internet-based interventions were administered to the experimental group without specification regarding dosage or a “use as you will” instruction [27,29,31,35,36,38], meaning that users could use the intervention whenever they wanted and how long they wanted. The other studies provided a set of explicit directions, such as requiring the user to log in at least once every week to view certain content [30,33] or for specific amounts of time [34,37]. Duration of interventions varied between 30 days [27] to 2 years [29,31,35]. Three control conditions were found within the included studies: (1) access to online resources (eg, e-bulletin on specific subjects, list of websites) [28,31,33-35], (2) access to selected portions of the experimental intervention [29,37], or (3) usual care/wait list [27,30,32,36,38]. Usual care for caregivers was either defined as the provision of information and education regarding care for the loved one [30,32] or not defined at all [31,33,36]. Adherence and usage were reported in six studies [27,30,31,33,34,36] and varied across interventions. For example, Kim et al [33] reported 100% adherence to requested usage (eg, all participants completed all the nine sessions planned in the program), whereas DuBenske et al [31] reported that 73% of the participants logged in at least once during the study.

As detailed in Table 3, the risk for selection bias was low for all the studies, and low to high for performance, detection, and attrition biases according to the tool of the Cochrane handbook [26]. Reasons for higher risk of bias concerned blinding of participants and outcome assessors, lack of control for co-intervention, and high rates of dropouts. Risk of bias in how participants performed and rated their health status at the time of completion was high overall, as only one study blinded their participants to group allocation [28] and one controlled for co-intervention [30]. A possibility for detection bias was also present for all studies because all assessments used for reporting the outcomes were based on self-report. This could have also led to a social desirability bias. Furthermore, the study of Beauchamp et al [27] did multiple testing without apparent statistical corrections, which can also lead to a detection bias. One study was judged to present a very high risk for detection bias because the outcome assessor was not blinded and the measures were administered during a face-to-face session [30]. As for the dropout rates, they were of 30% or more in both groups in five studies [29-31,35,36] and differed by 10% or more between the experimental and control groups in two studies [28,37]. Reasons for dropouts were always explored and missing data were treated with proper statistical analysis (eg, imputation techniques, intention-to-treat analysis, statistical models). Although these measures were taken, risk of bias for attrition was still judged high for these studies with large rates of dropouts and/or differential rates between experimental and control groups.

**Table 3:** Risk of bias.^a^

Author	Selection bias		Performance bias	Detection bias	Attrition bias
	Random sequence generation	Allocation concealment	Single blind	Blinding of outcome assessor	Missing data
Beauchamp et al [27]	+	+	–	+	+
Blom et al [28]	+	+	+	+	–
Chih et al [29]	+	+	–	+	–
Cristancho-Lacroix et al [30]	+	+	–	–	–
DuBenske et al [31]	+	+	–	+	–
Eames et al [32]	+	+	–	+	+
Kim et al [33]	+	+	–	?	+
McLaughlin et al [34]	+	+	–	+	+
Namkoong et al [35]	+	+	–	+	–
Pierce et al [36]	+	+	–	?	–
Smith et al [37]	+	+	–	+	–
Torkamani et al [38]	+	+	–	?	?

^a^+: low risk of bias; – high risk of bias; ?: unclear risk of bias.

### Categories and Components of Internet-Based Interventions

The included studies reported results concerning 10 interventions because the outcomes of one intervention were reported in three different articles [29,31,35]. Three intervention categories were found: Web-based education interventions [32,36,38], self-help Web-based therapeutic interventions [27,34], and human-supported Web-based therapeutic interventions [28-31,33,35,37]. There were neither online counseling activities nor Internet-operated therapeutic software in our sample. The components, as categorized by Barack and Klein [19], are presented in Table 4 and concern the use of multimedia, interactive online activities, and the provision of guidance and support, either automatically by the program or by a human (peer or professional).

#### Multimedia

All interventions used written text as their main multimedia component and some also used videos [27,28,30,33,34,37]. Videos were skill-based or educational; in the intervention by McLaughlin et al [34], the videos were designed to teach the caregivers a specific set of advocacy skills, whereas in the intervention by Kim et al [33] the videos were recorded lectures, supported by PowerPoint presentations, intended for the caregiver to watch and learn about various topics related to stroke.

#### Interactive Online Activities

Most interventions offered interactive online activities, either in the form of homework, quizzes, and exercises to reinforce the educational content [28,33,34,37], or in the form of online questionnaires [27,29,31,35,38]. In the intervention by Beauchamp et al [27], online questionnaires asked about caregiver status to tailor the intervention so that participants would view only relevant content for their situation. For example, selecting the “spouse” status revealed content about finances, socializing, and losing a companion. Similarly, online questionnaires about the caregiver and care recipient’s health status in a multicomponent intervention for caregivers of people with cancer [29,31,35] were used for tailoring. In this intervention, answers were also compiled by the program and shared with the clinical team in the form of a clinical report, including graphics visually representing the answers and the evolution in the dyad’s health status. It was also the only Internet-based intervention with a decision aid system, called the “coaching service,” which offered caregivers detailed action plans and instructions to change behavior based on a detailed analysis of the responses to online questionnaires.

#### Guidance and Supportive Feedback

Human support was given asynchronously (eg, forum, email, bulletin boards) [28-31,33,35-38] and synchronously (eg, live chat session) [37]. Human support was offered by a health professional (eg, nurse, clinician, or psychologist) in six interventions [28,29,31,33,35-38] to address questions and problems caregivers might have during the intervention. The intervention by Blom et al [28] offered professional feedback regarding caregiver homework and exercises, and participants could progress in the intervention only if they opened and checked the feedback. Five interventions also offered opportunities for peer support [29-31,35-38], with the intervention by Smith et al [37] being the only one using a group-support context with live chat sessions guided by a professional.

Other common components included the provision of links to additional resources [29,31,33-37] and written or videotaped testimonials of other caregivers [27,29-31,35].

**Table 4:** Component categories^a^ for each category of the Internet-based interventions.

Author		Multimedia	Interactive online activities^b^	Guidance and supportive feedback^b^	Other^b^
**Web-based education interventions**					
	Eames et al [32]	Text	NR	NR	NR
	Pierce et al [36]	Text	NR	Professional support: nurse specialist and rehabilitation team respond to questions with a private asynchronous module (email forum); peer support: asynchronous discussions facilitated by a nurse (email)	List of relevant Web links
	Torkamani et al [38]	Text	Online questionnaires on CR and CG health status	Professional support: clinicians receive answers from IOA, facilitating the speedy delivery of appropriate interventions; clinicians are also reachable with a “contact us” button; peer support: asynchronous discussion sessions (forum)	Musical entertainment; relaxation and exercise techniques
**Self-help Web-based therapeutic interventions**					
	Beauchamp et al [27]	Text; videos	Online questionnaires on CG personal situation; changing role button to select the relationship with CR	NR	IOA used to tailor content; testimonials
	McLaughlin et al [34]	Text; videos	Video-based skills exercises	NR	List of relevant Web links and articles
**Human-supported Web-based therapeutic interventions**					
	Blom et al [28]	Text; videos	Homework and exercises online; evaluation at the start and end of each lesson	Professional support: psychologist provides asynchronous feedback on IOA (electronic secured app); automatic reminders to send homework or attend lessons	Consultation of feedback is mandatory to have access to the next lesson
	Cristancho-Lacroix et al [30]	Text; videos lectures	NR	Peer support: asynchronous discussion sessions moderated by a psychologist (forum)	Relaxation training; testimonials; glossary; bank of activities to stimulate CR
	Chih et al [29]; DuBenske et al [31]; Namkoong et al [35]	Text; graphic	Online questionnaires on CR and CG health status; coaching service that automatically generates graphics of health status, offer decision aids, and structures an action plan	Professional support: cancer information specialist available via an “ask and expert” button.; Clinician report: summaries of users’ health available to the clinical team on demand, from a threshold alert or two days before a clinic visit; peer support: asynchronous discussion sessions moderated by a professional facilitator (bulletin board)	IOA and interactions through supportive feedback component used to tailor content; FAQs; list of relevant Web links, articles and community services; cancer news; testimonials ; personal webpage
	Kim et al [33]	Video lectures; PowerPoint slides	Online quizzes following the viewing of video lectures	Professional support: asynchronous service to network with health professionals (email)	List of relevant Web links
	Smith et al [37]	Text; video of enacted support group	At-home apps given by a nurse	Professional support: two times per week, a synchronous chat session directed by a nurse for the viewing and commenting of the weekly video (Adobe connect); the nurse is also available by asynchronous communication (email); peer support: asynchronous discussion sessions (email and message board)	List of relevant Web links, instructional videos and PDF files; online library of educational information; search engine

^a^As categorized by Barack and Klein [19].

^b^CG: caregiver; CR: care recipient; FAQ: frequently asked question: IOA: interactive online activities; NR: none reported.

### Behavior Change Techniques

All three Web-based education interventions used less than two BCTs [32,36,38] (Table 5). All the other interventions incorporated four to 10 BCTs each. Overall, 15 BCTs were used within our sample, out of the 26 possibilities of Abraham and Michie’s taxonomy [22]. The most commonly used technique was “social support” and this was offered through the component of peer support [29-31,35-38], except for one intervention that trained caregivers in planning social support instead of offering it to them [27]. Half of the interventions also provided a combination of two techniques: “providing instructions” and “prompting barrier identification” [27-31,34,35]. These instructions were given by multimedia components and/or reinforced by interactive online activities and professional support. “Stress management techniques” [28-31,34,35,37,38], “prompting practice of behavior” [30,34,37], and “modeling or demonstrating behavior” [27,28,34] were other common techniques used. The multicomponent intervention for caregivers of people with cancer [29,31,35] was the only intervention using “goal setting” and “action planning” techniques with their coaching service component. The intervention by Smith et al [37] incorporated the highest number of BCTs (n=10). Specifically, caregivers had to watch videos of enacted support group, which offered opportunities for social comparison and identification with role models. They also had to complete homework and report on their performance in subsequent discussion sessions to receive comments from professionals and peers, which prompted practice of behavior, self-monitoring, and provided feedback on performance.

**Table 5:** Behavior change techniques for each category of Internet-based interventions.

Author		Behavior change techniques^a^	Caregiver outcomes (ES)^b^
**Web-based education interventions**			
	Eames et al [32]	NR	NSSD in caregiver strain
	Pierce et al [36]	NR	NSSD in depression symptoms and satisfaction with life
	Torkamani et al [38]	Social support; stress management	NSSD in caregiver burden, occurrence of psychiatric, and/or behavioral problems, depressive symptoms, and quality of life
**Self-help Web-based therapeutic interventions**			
	Beauchamp et al [27]	Barrier identification; instructions; modeling; social support	↓ stress (0.5); ↑ intention to get support (0.3); ↓ caregiver strain (0.2); ↑ caregiver gain (0.2); ↓ depressive symptoms (0.2); ↓ state anxiety (0.2); ↑ self-efficacy (0.2); NSSD in the use of specific stress-reduction strategies
	McLaughlin et al [34]	Barrier identification; instructions; modeling; prompt practice; stress management	↑ skill application (1.01); ↑ intention to use (0.7); ↑ knowledge (0.67); NSSD in satisfaction with life
**Human-supported Web-based therapeutic interventions**			
	Blom et al [28]	Barrier identification; instructions; modeling; feedback on performance; stress management; time management	↓ symptoms of anxiety (0.48); ↓ depressive symptoms (0.26)
	Cristancho-Lacroix et al [30]	Information on behavior-health link and on consequences; barrier identification; instructions; prompt practice; social comparison; social support; stress management	↑ knowledge (0.79); NSSD in perceived stress
	Chih et al [29]; DuBenske et al [31]; Namkoong et al [35]	Information on behavior-health link and on consequences; barrier identification; instructions; goal setting; social support; stress management; time management	↓ negative mood at 6 and 12 months; ↓ caregiver burden at 6 months; ↑ bonding = ↑ active coping; NSSD for preparedness, physical burden, and in levels of disruptiveness
	Kim et al [33]	Information on behavior-health link and on consequences; instructions; feedback on performance	↑ caregiver mastery
	Smith et al [37]	Information on behavior-health link and on consequences; intention formation; instructions; self-monitoring of behavior; feedback on performance; prompt practice; social comparison; social support; identification to role models; stress management	↓ depression at 11 weeks and 1 month follow-up; NSSD in sense of mastery, self-esteem, and social support

^a^As categorized by Abraham and Michie [22].

^b^Arrows show the direction of statistically significant differences in intervention group compared to control for outcomes measured (*P*<.05). ES: value of effect sizes as originally reported by the authors; NR: none reported; NSSD: not statistically significant difference.

**Table 6:** Classification of the statistically significant outcomes by categories of Internet-based interventions and according to the Stress Process Model.^a^

Outcome		Web-based education interventions				Self-help Web-based therapeutic interventions			Human-supported Web-based therapeutic interventions				
		[32]	[36]	[38]	[27]		[34]	[28]		[30]	[29,31,35]	[33]	[37]
**Primary stressors**													
	Problematic behavior									0			
	Relationship quality									0^b^			
**Secondary role strains**													
	Disruptiveness										0		
**Secondary intrapsychic strains**													
	Caregiver gain				+								
	Mastery										0	+	0
	Self-esteem												0
**Outcomes**													
	Depression		0	0	+			+		0	+		+
	Anxiety				+			+					
	Stress				+^b^					0			
	Caregiver strain				+								
	Caregiver burden	0		0						0	+		
	Physical burden										0		
	Self-perceived health												
	Quality of life		0	0			0						
**Mediators**													
	Intention to get support				+^b^								
	Social support												0
	Coping				0					0^b^			
**Others**													
	Self-efficacy				+^b^					0^b^			
	Knowledge						+^b^			+^b^			
	Skill application						+^b^						
	Perceived bonding										+		

^a^+: Statistically significant effect (*P*<.05) of the intervention on the measured outcome (either improving positive factors or decreasing adverse factors); 0: not statistically significant effect.

^b^Validation process of the measure was not reported.

### Outcomes

A list of all the outcomes measured and statistically significant effects found at time of completion for each intervention is classified according to the Stress Process Model in Table 6. Outcomes were assessed with self-reported measures in all the included studies, either online [27-29,31,34,35], during phone calls [32,33,36,37], and/or face-to-face interviews [30,32,33,38]. All studies assessed outcomes at time of completion. Some also had an assessment half-way through the intervention [28,29,31,35,36,38] and two studies had a follow-up period of one [37] and six months [30].

Concerning outcomes at time of completion, none of the Web-based education interventions reported statistically significant differences on any outcomes when compared to usual care [32,36,38]. Efficacious interventions were thus found within the self-help and human-supported Web-based therapeutic interventions categories [27-31,33-35,37]. The most frequently assessed outcome was depression; it was shown as being significantly decreased by four interventions [27-29,31,37]. The intervention by Beauchamp et al [27] generated the largest number of positive effects on caregivers; in addition to decreased depression and anxiety, the intervention had positive effects on intrapsychic strains (eg, increase in caregiver gain), on mediators of stress (eg, intention to get support), and on self-efficacy. Namkoong et al [35] found that users of their multicomponent intervention, which integrated peer support, experienced a sense of bonding with the other participants, which in turn had a positive influence on mediators of stress such as coping abilities. In terms of longitudinal outcomes, neither of the two studies with a long-term follow-up showed statistically significant outcomes at these time points [30,37].

## Discussion

The goal of this study was to systematically review the best available evidence regarding the efficacy of Internet-based interventions for caregivers of older adults. Specifically, we sought to narratively synthesize the components integrated in such interventions following the classification of Barack and Klein [19] and the taxonomy of Abraham and Michie [22] to eventually link intervention components and BCTs with outcomes on caregivers’ stress process and well-being. Twelve studies with a high level of evidence covering 10 Web-based Internet interventions were found and analyzed in depth. A synthesis of the results in a comprehensive table is available online (Multimedia Appendix 2).

### Categories and Components of Internet-Based Interventions

Results from the review concerned Web-based education intervention, self-help Web-based therapeutic interventions, and human-supported Web-based therapeutic interventions. Online counseling, Internet-operated therapeutic software (including emerging technologies such as robotics, therapeutic gaming, and three-dimensional environments), and other online activities were not found in studies of a high level of evidence, which may reflect the novelty of research in these categories. Studies from these categories of Internet-based intervention are currently either at a pilot stage or have a lower level of evidence [39,54], which were not considered in this review.

Concerning the components, a combination of interactive online activities and provision of human support seemed to generally lead to better outcomes in caregivers. Exercises, homework, and questionnaires were the most used components from the interactive online activities’ category and appeared to be part of the success of the efficacious interventions. This can be explained by the fact that they linked to the use of BCTs and to the provision of human support. On one hand, exercises and homework were used to reinforce and build on the knowledge and skills caregivers learned while reading or viewing the content of the interventions, which can be viewed as the usage of “prompt practice” and “model behavior” techniques. In this way, Internet-based interventions represent a valuable advantage over telephone-based interventions or printed educational material [17] because they can enable participation to such interactive online exercises. On the other hand, results of online questionnaires were often sent to health professionals, which contributed to creating a continuous link between participants and clinicians. The ability to easily communicate with care providers and to monitor one’s own health or the health of the care recipient can be of high importance for remote caregivers, especially if they do not live with the care recipient [68]. Effective monitoring, with online questionnaires and planned professional support, also addresses a need for longitudinal assessment of caregiver outcomes, as recommended by guidelines for interventions for caregivers [7]. Furthermore, interactive online activities were used to tailor the content accessed by caregiver, thus rendering the intervention more meaningful and personal to every participant’s own needs and situation. Tailoring is an effective way to transmit content and to engage participants in an intervention [69,70], which can be easily done by Internet-based interventions with internal algorithms. Overall, interactive online activities may be used as an axle component that link different parts of the intervention to make it more appealing and engaging for the caregiver.

Human support, either provided by a health professional or peers, asynchronously or synchronously, was a component widely used in the interventions included and might account for the reported efficacy of human-supported Web-based therapeutic interventions. Having rapid and remote access to a health professional for advice and tailored support has been reported in previous studies of Internet-based interventions for care recipients as the primary factor predicting adherence [69,71] and positive effects on behavior change [20]. Qualitative results from previous research on Internet usage for professional support among caregivers suggest remote professional support was appreciated for the rapidity of the answers [47] and can alleviate barriers caregivers sometimes experience during face-to-face appointments (eg, feeling like a burden for the professional, hesitant to ask questions or express feelings) [72]. Knowing that they have access at any time and place to professional support may also make caregivers feel less worried [47,72]. Support from a group of peers was also found as a key factor of the efficacy of Internet-based interventions for older adults [71] and is reported as one of the three main reasons of using Internet among caregivers of people with cancer [73]. Furthermore, Kinnane and Milne [73] reported that caregivers viewed online support groups as means to communicate information about caring, planning for future steps to come, exchanging support in difficult moments, and venting feelings. Thus, peer support appears to be an effective component of BCT, possibly in relation to the fact that it can help support the development of effective coping strategies, which are important mediators of stress [9]. It is difficult to say if the way human support is provided (asynchronous vs synchronous) has any impact on the efficacy of human support itself because most of the included interventions used asynchronous human support. Asynchronous communication can cost less to developers and be used more easily by caregivers than complex synchronous communication modes, such as videoconference. However, in a study comparing a chat group to a video support group for caregivers of people with Alzheimer disease, caregivers reported feeling more at ease on the video group and experienced a more natural communication with the other caregivers, despite technical difficulties [44]. This sense of closeness and fluent conversation should not be understated and could have potential impact on caregiver outcomes, especially in interventions focusing on providing peer support. Overall, available evidence and results from this review suggest that human support should be considered as an efficacious component when designing Internet-based interventions.

### Behavior Change Techniques

Despite recommendations [20], integration of BCTs was not consistent across interventions. Indeed, little more than half of the 26 possible BCTs [22] were incorporated in the Internet-based interventions and concerned mostly the provision of “social support,” “instructions,” and “problem-solving techniques.” Yet, in a meta-analysis of Internet-based interventions promoting behavior change, Webb et al [20] found that interventions incorporating stress management had the greatest effect on behavior. Only four interventions used this technique in our sample [28-31,34,35] by offering caregivers concrete strategies to reduce stress, relaxation activities, or a detailed action plan to manage daily life and stressors. Moreover, results from the most recent national survey on caregiving in the United States show that stress management is the second information caregivers want to obtain, after information on how to maintain the care recipient at home [3]. Lastly, none of the three Web-based education interventions, which included only generic education content, had significant effects on any caregiver outcomes [32,36,38], which has also been reported by a review of Internet-based interventions for caregivers of people with dementia [11]. This can be explained by the lack of behavior change content and the use of very few to no BCTs to support the caregiver in achieving better health and well-being. Likely, traditional education interventions, delivered through printed material or face-to-face, have been shown to have little to no effect on caregivers’ outcomes [74]. In general, Internet-based interventions should incorporate more BCTs with possibly more focus on stress management techniques and contributing factors.

### Outcomes

The efficacy of Internet-based interventions for caregivers of older adults has been primarily demonstrated for psychological outcomes, such as a reduction in depression, anxiety, and burden, in this review and previous ones [11-14,16,17]. These results, although informative, permit only partial understanding of the underlying causes on which the interventions act. Indeed, in the Stress Process Model, depression, anxiety, and burden are only the outcomes of a long chain of primary and secondary stressors. Very few of the included interventions sought to measure more proximal indicators of well-being in the stress process, such as the quality of the relationship with the care recipient, disruptiveness, self-efficacy, and sense of mastery. Yet, these indicators are of clinical interest [7] and can prevent overexhaustion and serious mental difficulties if they are targeted earlier in the service delivery continuum. Furthermore, Internet-based interventions should be more concerned with enhancing mediators of stress, such as coping strategies and social support, as these are among the principal predictors of burden and decreased health for caregivers of older adults [75]. Finally, studies that measured quality of life did not show significant differences between groups at three months [34,36,38], possibly because these outcomes may not improve during a relatively short intervention.

### Research Gaps and Recommendations

Methodological and reporting differences in the studies limit the conclusions that can be currently drawn concerning Internet-based interventions for caregivers of older adults. Firstly, there was considerable heterogeneity in factors that can impact on the efficacy of the intervention, namely the dosage, the adherence, and the comfort of the users with the Internet. Only half of the studies reported usage metrics or adherence statistics, making it difficult to establish the frequency and the length of usage needed for an Internet-based intervention to reach full efficacy. Without knowing if participants adhered to instructions given, it is also difficult to draw conclusions about the feasibility of the intervention or whether it adds to the burden of care. To rectify this situation, intended use should be described and adherence should be carefully monitored throughout each trial and reported as a process outcome [69], with reasons for nonadherence detailed. This could inform future studies to determine the best delivery protocols for Internet-based interventions.

Secondly, chosen control conditions for all studies, except two [29,37], did not permit to exactly isolate which components were associated with efficacy. Rather, we can only hypothesize on which combination of components and factors can lead to better outcomes in caregivers. Although comparing Internet-based interventions to usual care or educational e-bulletin serves to demonstrate their combined efficacy, it does not permit isolation of the effects of each component specifically. The work of Chih et al [29] is a good example of how to obtain that information; the participants in the control group had access to the same Internet-based intervention (CHESS), but without the component of interest (eg, clinical reports). Isolation of the effects of each component could help future research provide better cost-benefit analyses because some components require more resources than others to develop. This could also help developers and decision makers in health care prioritize what components to incorporate in Internet-based interventions to maximize the efficacy of current services.

Thirdly, there were several methodological weaknesses within the studies that reduced the validity of their findings, namely lack of blinding, high rates of attrition, uncontrolled risks for co-intervention, and unclear reporting. Blinding is a difficult criterion to fulfill within psychosocial intervention trials, but not impossible as demonstrated by Blom et al [28]. Future Internet-based trials should do likewise to reduce bias in reported outcomes. Unsurprisingly, trials that targeted caregivers of people with terminal or degenerative conditions (eg, cancer, dementia) registered higher levels of attrition, mainly due to the death of the care recipient or the overwhelming burden of the caregiver. Some studies did not clearly report critical information, which made it difficult to determine the validity of methods employed. Following guidelines for reporting Internet-based trials, such as the CONSORT-EHEALTH guidelines [25], could improve reporting and increase the confidence in the findings.

Finally, with the current sample it is not possible to determine if positive effects of Internet-based interventions are maintained over time. Indeed, measures were mainly taken on completion of programs and only two studies had a moderate to long-term follow-up period (one and six months) [30,37]. Future trials could document long-term outcomes to compare the efficacy of Internet-based interventions to traditional face-to-face interventions over time.

### Limitations and Strengths

In terms of methods, there are several limits to this systematic review. First, a meta-analysis of the data was not performed given the heterogeneity of the outcomes, which restricts the findings to the state of hypotheses. Second, initially included studies were not counterverified by a second author. To ensure that we would capture the best evidence available despite this weakness, the research strategy was expanded to all possible wording of keywords of interest. Uncertainty concerning the inclusion of a study was always resolved with a second or third opinion. Therefore, we are confident that this systematic review covers the best evidence currently available in the field of Internet-based interventions for caregivers of older adults. Third, coding of the included interventions was performed by only one author and relied on the information reported by the authors in the studies, which might not adequately represent all the components of the delivered intervention. A hallmark of this review was the use of appropriate categorizations to describe and analyze the Internet-based interventions and the use of BCTs. This proved relevant in comparing different interventions with varying levels of interactivity and guidance, which helped to draw useful conclusions.

### Conclusions

The findings from this systematic review suggest that Internet-based interventions with tailored behavior change content that are interactive, provide human support either by professionals or peers, and incorporate BCTs, such as provision of specific instructions regarding the behavior, problem solving, and stress management, can have positive effects on the psychological well-being of caregivers of older adults. Further randomized controlled trials that demonstrate the effect of each component individually with appropriate control conditions, analyze their outcomes considering adherence to protocol, and structure their report according to reporting guidelines in eHealth are needed to strengthen the validity of these results.

## Appendix

Multimedia Appendix 1 MEDLINE MeSH terms and keywords.

Multimedia Appendix 2 Synthesis of components, behaviour change techniques and outcomes for each category of Internet-based interventions.

## Abbreviations

BCTs: behavior change techniques
CONSORT: Consolidated Standards of Reporting Trials
GRADE: Grading of Recommendations Assessment, Development and Evaluation
PRISMA: Preferred Reporting Items for Systematic Reviews and Meta-Analyses
