# Supplementary material for: Components and Outcomes of Internet-Based Interventions for Caregivers of Older Adults: Systematic Review
Source: J Med Internet Res. 2017 Sep 19;19(9):e313. doi: 10.2196/jmir.7896 (PMC5627044; doi:10.2196/jmir.7896)
Supplement: Multimedia Appendix 1 [file jmir_v19i9e313_app1.pdf]

Multimedia Appendix 1 MEDLINE MeSH terms and keywords

1. caregivers/
2. spouses/
3. siblings/
4. family/
5. caregiv*.mp.
6. care-giv*.mp.
7. informal care.mp.
8. carer*.mp.
9. spouse*.mp.
10. family caregiv*.mp.
11. (sibling*or brother* or sister*).mp.
12. aged/
13. “aged, 80 and over”/
14. Frail Elderly/
15. (aged or elder* or geriatr* or senior* or old or older).mp.
16. social support/
17. self care/
18. self concept/
19. patient education as topic/
20. (psychosocial support* or social support* or support system* or support network* or social network*).mp.
21. (self car* or selfcar*).mp.
22. self manag*.mp.
23. self efficacy.mp.
24. (behavior chang* or behaviour chang* or behaviour modification* or behaviour modification*).mp.
25. Internet/
26. blogging/
27. social media/
28. mobile applications/
29. electronic mail/
30. telemedicine/
31. remote consultation/
32. cell phones/
33. text messaging/
34. microcomputers/
35. computers, handheld/
36. Computers/
37. Computer Communication Networks/or Software/
38. mobile.mp.
39. (cell* phone* or cell* telephone* or mobile phone* or mobile telephone*).mp.
40. (handheld or hand-held).mp.
41. microcomput*.mp.
42. (pda* or personnal digital assistant*).mp.
43. (smartbook or smart-book or smartphone* or smart phone* or tablet).mp.
44. (portable and (comput* or technolog*)).mp.
45. (website* or web* or web-based or webbased or webpage).mp.
46. (app or apps or mobile application*).mp.
47. (electronic mail* or e-mail* or email*).mp.
48. Internet.mp.
49. (messag* or text messag* or sms or short message service).mp.
50. (skyp*or video* or multimedia or youtube).mp.
51. (telemed* or telemonitor* or telepsych* or telecar* or telehealth).mp.
52. (ehealth or e-health).mp.
53. (virtual* or digital*).mp.
54. (blog* or vlog*).mp.

1 or 2 or 3 or 4 or 5 or 6 or 7 or 8 or 9 or 10 or 11 = 55

16 or 17 or 18 or 19 or 20 or 21 or 22 or 23 or 24 = 56

25 or 26 or 27 or 28 or 29 or 30 or 31 or 32 or 33 or 34 or 35 or 36 or 37 or 38 or 39 or 40 or 41 or 42 or 43 or 44 or 45 or 46 or 47 or 48 or 49 or 50 or 51 or 52 or 53 or 54 = 57

55 and 56 and 57

limit 58 to (yr="2000 -Current” and (english or french))

/, exact MeSH term; * wildcard; mp., search in multiple places.
