# Supplementary material for: Components and Outcomes of Internet-Based Interventions for Caregivers of Older Adults: Systematic Review
Source: J Med Internet Res. 2017 Sep 19;19(9):e313. doi: 10.2196/jmir.7896 (PMC5627044; doi:10.2196/jmir.7896)
Supplement: Multimedia Appendix 2 [file jmir_v19i9e313_app2.pdf]

**Multimedia appendix 2** Synthesis of components, behaviour change techniques and outcomes for each category of Internet-based interventions

| Author | Categories of components [a] | | | | Behaviour change techniques [c] | Caregiver outcomes (ES) [d] |
|---|---|---|---|---|---|---|
| | Multi-media | IOA [b] | Guidance and supportive feedback [b] | Other [b] | | |
| Web-based education interventions | | | | | | |
| Eames et al [32] | Text | NR | NR | NR | NR | NSSD in caregiver strain |
| Pierce et al [36] | Text | NR | *Professional support:* nurse specialist and rehabilitation team respond to questions with a private asynchronous module (email forum) *Peer support:* asynchronous discussions facilitated by a nurse (email) | List of relevant web links | NR | NSSD in depression symptoms and satisfaction with life |
| Torkamani et al [38] | Text | Online questionnaires on CR and CG health status | *Professional support*: clinicians receive answers from IOA, facilitating the speedy delivery of appropriate interventions. Clinicians are also reachable with a "contact us" button. *Peer support:* asynchronous discussion sessions (forum) | Musical entertainment Relaxation and exercise techniques | Social support Stress management | NSSD in caregiver burden, occurrence of psychiatric and/or behavioral problems, depressive symptoms and quality of life |

| | | | | | | |
|---|---|---|---|---|---|---|
| Self-help web-based therapeutic interventions | | | | | | |
| Beauchamp et al [27] | Text<br>Videos | Online questionnaires on CG personal situation<br>Changing role button to select the relationship with CR | NR | IOA used to tailor content<br>Testimonials | Barrier identification<br>Instructions<br>Modelling<br>Social support | ↓ stress (0.5)<br>↑ intention to get support (0.3)<br>↓ caregiver strain (0.2)<br>↑ caregiver gain (0.2)<br>↓ depressive symptoms (0.2)<br>↓ state anxiety (0.2)<br>↑ self-efficacy (0.2)<br>NSSD in the use of specific stress-reduction strategies |
| McLaughlin et al [34] | Text<br>Videos | Video-based skills exercises | NR | List of relevant web links and articles | Barrier identification<br>Instructions<br>Modelling<br>Prompt practice<br>Stress management | ↑ skill application (1.01)<br>↑ intention to use (0.7)<br>↑ knowledge (0.67)<br>NSSD in satisfaction with life |
| Human-supported web-based therapeutic interventions | | | | | | |
| Bloom et al [28] | Text<br>Videos | Homework and exercises online<br>Evaluation at the start and end of each lesson | *Professional support:* psychologist provides asynchronous feedback on IOA (electronic secured application)<br>*Automatic reminders* to send homework or attend lessons | Consultation of feedback is mandatory to have access to the next lesson | Barrier identification<br>Instructions<br>Modelling<br>Feedback on performance<br>Stress management<br>Time management | ↓ symptoms of anxiety (0.48)<br>↓ depressive symptoms (0.26) |

| | | | | | | |
|---|---|---|---|---|---|---|
| Cristancho-Lacroix et al [30] | Text<br>Videos lectures | NR | *Peer support:* asynchronous discussion sessions moderated by a psychologist (forum) | Relaxation training<br>Testimonials<br>Glossary<br>Bank of activities to stimulate CR | Information on behaviour-health link and on consequences<br>Barrier identification<br>Instructions<br>Prompt practice<br>Social comparison<br>Social support<br>Stress management | ↑ knowledge (0.79)<br>NSSD in perceived stress |
| Chih et al [29]; DuBenske, et al [31]; Namkoong et al [35] | Text<br>Graphic | Online questionnaires on CR and CG health status<br>Coaching service that automatically generates graphics of health status, offer decision aids and structures an action plan | *Professional support:* cancer information specialist available via an “ask and expert” button.<br>*Clinician report:* summaries of users’ health available to the clinical team on demand, from a threshold alert or two days before a clinic visit.<br>*Peer support:* asynchronous discussion sessions moderated by a professional facilitator (bulletin board) | IOA and interactions through supportive feedback component used to tailor content<br>FAQs<br>List of relevant web links, articles and community services<br>Cancer news<br>Testimonials<br>Personal webpage | Information on behaviour-health link and on consequences<br>Barrier identification<br>Instructions<br>Goal setting<br>Social support<br>Stress management<br>Time management | ↓ negative mood at 6 and 12 months<br>↓ caregiver burden, at 6 months<br>↑ bonding = ↑ active coping<br>NSSD for preparedness, physical burden<br>and in levels of disruptiveness |
| Kim et al [33] | Video lectures<br>Power points | Online quizzes following the viewing of video lectures | *Professional support:* asynchronous service to network with health professionals (email) | List of relevant web links | Information on behaviour-health link and on consequences<br>Instructions<br>Feedback on performance | ↑ caregiver mastery |

| | | | | | | |
|---|---|---|---|---|---|---|
| Smith et al [37] | Text<br>Video of enacted support group | At-home applications given by a nurse | *Professional support:* two times per week, a synchronous chat session directed by a nurse for the viewing and commenting of the weekly video (Adobe connect).<br>The nurse is also available by asynchronous communication (email).<br>*Peer support:* asynchronous discussion sessions (email and message board) | List of relevant web links, instructional videos and pdf files.<br>Online library of educational information<br>Search engine | Information on behaviour-health link and on consequences<br>Intention formation<br>Instructions<br>Self-monitoring of behaviour<br>Feedback on performance<br>Prompt practice<br>Social comparison<br>Social support<br>Identification to role models<br>Stress management | ↓ depression, at 11 weeks and one month follow-up<br>NSSD in sense of mastery, self-esteem and social support. |

[a] As categorized by Barack and Klein [19].

[b] CG: caregiver; CR: care recipient; FAQ: frequently asked question: IOA: interactive online activities; NR: none reported.

[c] As categorized by Abraham and Michie [22].

[d] Arrows show the direction of statistically significant differences in intervention group compared to control for outcomes measured (P<.05). ES: value of effect sizes as originally reported by the authors; NSSD: non statistically significant difference.
